# Social isolation, social media use, and poor mental health among older adults, California Health Interview Survey 2019–2020

**DOI:** 10.1007/s00127-023-02549-2

**Published:** 2023-09-20

**Authors:** Hafifa Siddiq, Senait Teklehaimanot, Ariz Guzman

**Affiliations:** 1 Charles R. Drew University of Medicine and Science, Los Angeles, USA; 2 Cedars-Sinai Medical Center, Los Angeles, USA

**Keywords:** Loneliness, Mental health, Older adults, Social media use, Social isolation

## Abstract

**Background:**

Older adults’ engagement on social media may be a resource to reduce loneliness and improve mental health. Purpose: This study aimed to examine loneliness and social media use and its association with severe psychological distress among older adults and whether social media use moderated the association of loneliness on severe psychological distress among older adults.

**Methods:**

This study is a secondary analysis of the 2019–2020 California Health Interview Survey. The Kessler 6-item Psychological Distress Scale (K6) assessed symptoms of psychological distress, with a K6 score ≥ 13 associated with severe mental illness. Loneliness was measured using a revised Three Item Loneliness Scale (TILS) score. Multiple regression analyses were conducted to examine associations between loneliness and severe psychological distress.

**Results:**

Loneliness, health status, and identification as Asian, remained strong predictors of severe psychological distress among older adults when adjusting for other factors. In bivariate analysis, high-frequent social media users, but did not remain significant when accounting for covariates. Social media use did not moderate the association between loneliness and severe psychological distress.

**Conclusion:**

This study underscores the significant impact of loneliness on poor mental health among older adults, emphasizing that while frequent social media use correlates with severe psychological distress, it may not alleviate the association of loneliness on poor mental health, thus highlighting the urgent need to address social isolation and promote genuine social connectedness, particularly among Asian older adults.

**Supplementary Information:**

The online version contains supplementary material available at 10.1007/s00127-023-02549-2.

## Introduction

Social isolation and loneliness are significant public health and policy concerns. Due to the restrictive social distancing policies and a general sense of uncertainty during the COVID-19 outbreak, the population’s mental health has been subject to a worldwide growing concern [7, 17]. The significant negative consequences social isolation has on longevity, mental and physical health, and well-being, are well-documented [4, 32]. According to advisory from the United States (U.S.) Surgeon General, social isolation can increase the risk for premature death to levels comparable to smoking 15 cigarettes a day [12]. From a life-course perspective, older adults represent a vulnerable population with a heightened risk for loneliness and social isolation [32]. There is a need to understand strategies to promote social connectedness, particularly the role of social media use, that may address loneliness and promote the mental health of older adults within the pandemic context [11].

Social isolation has well-documented detrimental effects on health and well-being. Subjective social isolation, or loneliness, is defined as “the individual’s dissatisfaction with the frequency and closeness of their social contacts or the discrepancy between the relationships they have and the relationships they would like to have” [41]. Loneliness has emerged as a significant risk factor for adverse health outcomes and consistently linked to poor mental health [28, 32]. Studies have demonstrated strong associations between loneliness and conditions such as depression, suicidal ideation, moderate to severe psychological distress, and even premature death [25, 37]. Moreover, a longitudinal study highlights loneliness as a predictor of increased depression, anxiety, and overall mental health problems over time [34]. A meta-analysis further supports these findings, revealing a pooled adjusted odds ratio of 2.33 (95% CI 1.62–3.34) for the risk of new onset depression among adults who experience frequent loneliness compared to those who do not [25].

To date, limited research has explored racial and ethnic disparities in loneliness and poor mental health [42]. However, emerging evidence sheds light on this critical issue. Data from the Mental Health Foundation reveals that approximately one in three Black individuals have experienced loneliness at a higher rate than the general population [1]. Moreover, research by Miyawaki [30] indicates that perceived isolation significantly negatively impacts mental health in Black and Hispanic individuals. This study also finds that Black older adults experience significantly higher rates of perceived social isolation and worse self-rated mental health compared to their White counterparts [30]. A more recent study using data from the Health and Retirement Study (HRS) reveals that race significantly moderates the relationship between loneliness and depressive symptoms when controlling for sociodemographic covariates, social support, negative interaction variables, and religious service attendance [42]. These findings suggest that experiences of racism, discrimination, and social exclusion may contribute to higher rates of social isolation, loneliness, and poor mental health among racial and ethnic minority individuals. Given these associations, racial and ethnic minority older adults may be particularly vulnerable to the adverse impacts of loneliness and social exclusion on mental health [8, 14].

Social media and technology have the potential to foster social connectedness and alleviate loneliness, although older adults traditionally have lower rates of social media usage. Regardless, the use of social media has become increasingly prevalent as a means of promoting social engagement and interaction [15]. Social theories suggest that social media use can influence individuals’ perceptions of, maintenance of, and interaction with their social networks, which, in turn, can impact mental health outcomes [27]. Despite their initially lower adoption rates, older adults have shown increasing enthusiasm for embracing new networking tools, including social media [3]. This indicates the potential of social media as a vital resource for social engagement, particularly during challenging times such as the pandemic, when social distancing, self-isolation, or quarantine measures have been implemented [38].

The impact of social media use on the mental health of populations, especially among older adults, remains a topic of ongoing debate with no clear consensus [16]. Some studies suggest potential positive effects of social technology use among older adults, linking it to better self-rated health, fewer chronic illnesses, higher subjective well-being, and reduced depressive symptoms [2]. Other positive consequences of social media use may help to overcome loneliness, relieve stress, and raise feelings of control and self-efficacy [22]. However, evidence from systematic reviews on social media and mental health in the general population reveals mixed findings, with both positive and negative associations with anxiety and depression [16]. It is important to note that the existing research is not exhaustive, as few randomized controlled trials specifically focus on the role of social media in mental health, particularly among older adults [40]. Most studies on social media use tend to concentrate on adolescent and young adult populations.

Social media has many positive benefits, but constant use may also lead to adverse mental health outcomes like depression, anxiety, and suicidality—especially among younger-aged groups and vulnerable populations [23, 26, 44]. A cross-country analysis of 39 studies found that while social media can promote social connections and community, excessive use of social media is correlated with depression and other mental health disorders [44]. Studies also suggest that prolonged use of social media platforms may relate with negative signs and symptoms of depression, anxiety, and stress [33]. Similarly, a National Survey of Adults over 45 on Loneliness and Social Connections finds that using technology for communication has a small but significant effect on loneliness—as people use social media more, they report feeling more lonely [1]. While the literature is mixed, the benefits of social media include the facilitation of better interpersonal relationships and socialization for older adults and warrants further exploration.

In light of the mixed literature and gaps in the present literature, this study seeks to expand upon previous research by examining the role of loneliness and social media use on poor mental health, using a large state-level and population-representative sample of older adults in California. This study has the following aims:

1. To examine the association between loneliness and social media use with poor mental health (measured as psychological distress) among adults over the age of 65. We hypothesize that loneliness and social media use will be significantly associated with psychological distress.
2. To examine the relationship between loneliness on poor mental health when accounting for covariates. We hypothesize that higher levels of loneliness will be a significant predictor of severe psychological distress.
3. To examine the role of level of social media use as a moderating factor between loneliness and poor mental health. We hypothesize that constant social media users over the age of 65 will have lower levels of loneliness and lower levels of psychological distress.

## Methods

This study is a secondary analysis of the 2019–2020 California Health Interview Survey (CHIS), the largest state health survey in the US and examines public health and health care access issues in California. CHIS is a cross-sectional, mixed-mode (web and telephone) survey that uses an address-based sampling frame to recruit study participants. For all sampled households, one randomly selected adult in each sampled household either completed an on-line survey or was interviewed by telephone. By using an address-based sampling frame, it is representative of the state's population. Surveys are administered in six languages: English, Spanish, Chinese (Mandarin and Cantonese dialects), Vietnamese, Korean, and Tagalog. Participants in the survey are randomly selected. The sample includes both households with landlines and those with cell phones only. To produce population estimates from CHIS data, weights were applied to the sample data. Only CHIS data among adults over the age of 65, were included in this study (N = 8447).

### Measures

#### Dependent variable: psychological distress

Prevalence of symptoms of serious psychological distress in the overall sample were measured using the Kessler 6 Psychological Distress Scale and categorized as none, low, moderate, and severe. Our main dependent variable uses a validated measure of severe psychological distress defined as a score of 13 or higher on the 0- to 24-point scale [10, 18]. Moderate level psychological distress was defined as a score between 8 and 12, and low-level psychological distress defined as a score between 1 and 7. Items are reverse coded so that cases with a greater frequency of symptoms receive higher scores. Scores are assigned based on the following criteria: all of the time (assigned score of 4), most of the time (assigned score of 3), some of the time (assigned score of 2), a little of the time (assigned score of 1), and not at all (assigned score of 0). The overall psychological distress value is the total of the assigned scores for the above items. Our main dependent variable dichotomizes severe psychological distress (defined as a score of 13 or above) or not severe. The criterion of greater than 13 has been shown to predict serious mental illness and is considered severe psychological distress by others [10, 29, 35].

#### Main independent variable: loneliness

Loneliness was captured using the revised UCLA loneliness scale to a Three Item Loneliness Scale (TILS) score [39]**.** The response categories to three separate questions were coded 1 (*hardly ever*), 2 (*some of the time*), and 3 (*often*). Each person's responses to the questions are summed, with higher scores indicating greater loneliness. A study reports the internal consistency for a three-item scale is quite good and indicates that the items reliably measure moderate to severe levels of loneliness [13]. The TILS score was dichotomized and defined those whose score across items was 1.5 or greater (equivalent to a summed score of 5 or greater for a score ranging from 3 to 9) as being “more lonely.” A summed score of corresponds to a “some of the time” response for at least 2 items or an “often” response for at least 1 item [9].

#### Moderator variable: level of social media

Social media use was measured using a question asking how often they used a computer for social media, with responses as: almost constantly, many times a day, few times a day, less than a few times a day.

#### Covariates: sociodemographic variables

Based on prior research on loneliness and mental health among older adults, factors including sociodemographic characteristics: self-reported gender (male or female), educational attainment (less than high school, some college, college degree, graduate degree and above), employment status (employed and unemployed), health insurance (yes or no), and health status (excellent, good/very good, or fair/poor) were included in this study. Race and ethnicity were categorized according to the California Department of Finance race/ethnicity classification specified by the federal Office of Management and Budget as non-Hispanic White, Hispanic/Latinx, non-Hispanic African-American, non-Hispanic Asian, and All other. Socioeconomic status was measured using poverty level (family income below the poverty line at 0–99%, 100–199%, 200–299%, and over 300%).

### Statistical analysis

Descriptive analysis was performed for all included variables. Each independent variable was analyzed using bivariate analysis. We also tested whether factors were associated with psychological distress when accounting for socio-demographic characteristics, using multivariate logistic regressions. Odds ratios and 95% confidence limit (CI) were calculated for each measure. All analyses accounted for complex survey design and weighting to produce state-representative findings, and were conducted using STATA 17.0.

## Results

Table 1 displays overall population characteristics of the sample of older adults in California by level of psychological distress. Among the sample of older adults in California (N = 8507), 57.3% were between the ages of 65 and 74, 32% were between the ages of 75 and 84, and about 10.7% were over the age of 85 (mean age = 71.8). Among the total sample of older adults, 59.8% were non-Hispanic White, 20.5% were Hispanic/Latinx, 10.9% were African American, 6.1% were Asian, and 2.6% were of other ethnicities. Over 13% reported severe levels of loneliness. Approximately 15.9% of the sample of older adults reported moderate to severe level of psychological distress. The sample reported overall low K6 scale scores with a weighted mean K6 scale score of 2.41.

**Table 1:** Population characteristics of adults over the age of 65 in California by level of psychological distress (N = 8507)

Variable	Category	Total (%)	No (34.1%)	Low (49.9%)	Moderate (11.9%)	Severe (4.0%)	p value (< 0.05)
Gender	Male	4076 (45.2)	1497 (37.9)	2076 (48.8)	390 (10.1)	107 (3.2)	0.003
	Female	4431 (54.8)	1328 (31.0)	2461 (51.0)	497 (13.3)	145 (4.7	
Age	65–74	5165 (57.3)	1659 (33.1)	2773 (50.6)	564 (12.2)	168 (4.1)	0.187
	75–84	2587 (32.0)	911 (36.5)	1370 (49.7)	240 (10.0)	63 (3.8)	
	> = 85	755 (10.7)	255 (32.7)	394 (47.2)	83 (15.3)	21 (4.7)	
Race/ethnicity	NH, White	6625 (59.8)	2125 (32.8)	3658 (53.7)	659 (10.4)	178 (3.1)	0.0001
	Hispanic/Latinx	736 (20.5)	248 (32.0)	347 (45.1)	113 (19.2)	28 (3.7)	
	NH, African American	297 (6.1)	121 (38.9)	144 (50.5)	22 (6.4)	9 (4.2)	
	NH, Asian	657 (11.0)	264 (41.5)	293 (9.5)	71 (9.5)	29 (9.3)	
	All other	192 (2.6)	67 (38.8)	95 (45.4)	22 (10.4)	8 (5.4)	
Education	< = High school	1438 (39.4)	481 (31.8)	685 (47.0)	195 (15.1)	74 (6.1)	0.0001
	Some college	2653 (22.6)	872 (34.7)	1395 (49.9)	293 (11.9)	91 (3.5)	
	College or some graduate school	2516 (22.0)	859 (36.4)	1358 (52.9)	245 (8.9)	53 (1.9)	
	Graduate degree or above	1900 (16.0)	613 (35.9)	1099 (53.3)	154 (7.9)	34 (2.8)	
Marital status	Married	4349 (56.2)	1558 (37.1)	2329 (50.4)	373 (10.0)	86 (2.5)	0.0001
	Widow/separated/divorced	3589 (38.8)	1110 (30.1)	1904 (49.9)	439 (14.1)	133 (5.9)	
	Living with partner Single/never married	569 (5.0)	157 (32.0)	304 (45.8)	75 (15.0)	33 (7.2)	
Insurance status	Yes	8490 (99.3)	2821 (34.3)	4531 (50.3)	881 (11.4)	251 (4.1)	
	No	17 (0.7)	4 (10.7)	6 (10.9)	6 (77.3)	1 (1.0)	
Employment status	Full-time	1124 (12.8)	372 (33.0)	621 (51.3)	106 (12.0)	25 (3.7)	0.0001
	Part-time	712 (7.7)	236 (37.3)	406 (48.8)	56 (10.9)	14 (3.1)	
	Unemployed	6671 (79.5)	2217 (34.0)	3510 (49.9)	725 (11.9)	213 (4.2)	
Poverty level	< 200 FPL	1670 (28.5)	468 (28.0)	830 (47.1)	256 (16.6)	113 (8.3)	0.0001
	> = 200% FPL	6837 (71.5)	2357 (36.6)	3707 (51.1)	631 (10.0)	139 (2.3)	
Health status	Excellent	4501 (44.8)	1842 (44.6)	2390 (48.4)	234 (6.3)	34 (0.7)	0.0001
	Good/very good	2620 (34.0)	754 (31.9)	1462 (53.4)	331 (11.6)	70 (3.0)	
	Fair/poor	1386 (21.2)	229 (15.3)	685 (47.8)	322 (24.0)	148 (12.9)	
Loneliness (TILS score)	Less lonely (3–5)	7340 (86.8)	2598 (39.9)	3534 (52.0)	362 (7.1)	43 (0.9)	0.0001
	More lonely (> 6)	1107 (13.2)	209 (13.8)	974 (42.7)	522 (28.5)	205 (15.0)	
Social media use	Almost always	162 (2.1)	56 (41.2)	60 (16.0)	33 (16)	13 (14.7)	0.0001
	Many times	1117 (12.7)	324 (30.5)	132 (13)	132 (13.0)	39 (5.1)	
	A few times	1994 (24.0)	618 (33.2)	200 (11.3)	200 (11.3)	60 (2.8)	
	< A few times	5228 (61.2)	1827 (35.0)	522 (11.7)	522 (11.7)	140 (3.9)	

Source: 2019–2020 California Health Interview Survey

Results from the bivariate analysis show that identifying as female, Asian, single or never married, living 200% above the poverty level, having fair or poor health status, loneliness, and use of social media were significantly associated with poor mental health (p < 0.05). Findings from this bivariate analysis support our first hypothesis that loneliness is associated with poor mental health among older adults. See Table 2 for results from bivariate and multivariate analysis.

**Table 2:** Bivariate and multivariable logistic regression analysis: relationship between independent variables and severe psychological distress among older adults (N = 8447)

Variables	Level/category	Bivariate analysis Crude OR [95% CI]	Multivariate analysis Adjusted OR [95% CI]
Gender	Male	Ref	Ref
	Female	1.81(1.04–3.14) *	1.03(0.52–2.06)
Race/Ethnicity	NH, White	Ref	Ref
	Hispanic	1.78(0.61–5.20)	0.99(0.34–2.85)
	NH, African American	2.40 (0.73–0.78)	3.00(0.82–10.94)
	NH, Asian	4.26(2.29–7.95)**	2.04(1.08–3.87)*
	All other	1.74(0.14–21.26)	0.58(0.03–11.61)
Educational Attainment	Graduate degree or above	Ref	Ref
	High school or less	1.76(0.74–4.18)	0.55(0.23–1.31)
	Some college	0.62 (0.24–1.55)	0.31(0.12–0.84)*
	College degree/some graduate school	0.46(0.20–1.06)	0.34(0.14–0.78)*
Marital status	Married	Ref	Ref
	Living with partner	1.37(0.42–4.49)	0.37(0.1–1.8)
	Widow/separated/divorce	2.7(1.80–3.9)**	1.22(0.17–2.61)
	Single/never married	3.30(1.82–6.0)**	0.66(0.17–2.61)
Employment status	Employed full-time	Ref	Ref
	Employed part-time	2.47(0.22–27.73)	1.13(0.1–10.80)
	Unemployed	2.31(0.56–9.43)	0.83(0.14–4.64)
Poverty level	< = 200% FPL	Ref	Ref
	> 200% FPL	4.92 (2.89–8.38)***	1.61(0.89–2.92)
Health status	Excellent	Ref	Ref
	Good	2.84(1.35–5.95)**	1.64(0.78–3.45)
	Fair/Poor	23.41(10.39–52.72)***	9.71(3.9–24.13)***
Loneliness (TILS score)	Less Lonely (3/5)	Ref	
	More Lonely (6/9)	27.02(14.23–51.29)***	23.11(10.75–49.71)***
Social media use	Almost always	Ref	Ref
	Many times	3.36(1.44–7.85) ***	6.14(0.01–0.254)
	A few times	0.58(0.22–1.54)	1.41(0.12–16.45)
	Less than few times	0.48(0.26–0.92)	0.93(0.16–16.45)

Source: 2019–2020 California Health Interview Survey

*< 0.05

**< 0.01

***< 0.001

Results from the multivariate analysis for psychological distress, which accounts for sociodemographic variables including loneliness and social media use, show five factors contributing to the overall model. Loneliness remained a significant predictor to poor mental health, supporting our second research hypothesis testing the predictive relationship between loneliness with mental health when accounting for covariates. Non-Hispanic Asian identity, [adjusted odds ratio (AOR) = 2.04, 95% confidence limit (95% CI) 1.08, 3.87, p < 0.05], having fair or poor health [AOR = 9.71, 95% CI 3.9, 24.13, (p < 0.001)], and loneliness [AOR = 23.11, 95% CI 10.75, 49.71, (p < 0.001)] had higher odds of severe psychological distress. Having some college-level education [AOR = 0.31, 95% CI 0.11, 0.83, (p < 0.05)], or a college degree or above [AOR = 0.33, 95% CI 0.33, 0.14, (p < 0.05)], appear to have protective association on the mental health of older adults. This analysis also tests the interaction term between social media use and mental health (Appendix). When accounting for covariates, including health status, social media use was not a significant predictor of mental health and social media use does not moderate the relationship between loneliness and psychological distress. This does not support our third hypothesis.

## Discussion

This study demonstrates that identifying as female, Asian, single, poor socioeconomic status, loneliness, and high level of social media use is significantly associated with severe psychological distress among California older adults. These findings indicate key factors for public health interventions addressing the mental health of older adult populations.

### Loneliness and mental health

Loneliness is a significant predictor for severe psychological distress among older adults, corroborating existing research examining loneliness and poor mental health outcomes like depression and anxiety and other mental health disorders in this population. This relationship remained statistically significant when accounting for sociodemographic characteristics and health status, supporting our second hypothesis. These findings indicate that although some sociodemographic factors may contribute to loneliness and psychological distress, both loneliness and health status exerts an influence on psychological distress that are independent of the influence of these sociodemographic factors. These findings are consistent with previous work on the detrimental effects of loneliness on the mental health of older adults [28, 37] and how subjective social isolation is associated with more severe psychological distress [43].

### Social media use and mental health

Our other objective was to investigate how an important coping resource with social isolation—level of social media use—may modify the relationship between loneliness and poor mental health. Additionally, we examined the relationship between loneliness on poor mental health among older adults, and whether this association differs for different levels of social media use. Our results were surprising**,** in the sense that while constant social media use was itself associated with older adults’ psychological distress, it did not moderate the relationship between loneliness and mental health. Social media use at limited or lower levels as a source of social support for older adults, may improve association between loneliness and poor mental health, and warrants further research.

Mixed findings of how social media use may influence the association between loneliness and poor mental health may be due to the large variability in how social media use is conceptualized in research. Studies that controlled for health covariates shows social media to have a non-significant association to depression [6]. However, it is important to include covariates critical to aging research which include other factors not accounted for in this study (like network characteristics, social support, chronic health conditions or comorbidities, and other health-related behaviors). Frequency and duration of social media use as a measure have been used primarily to quantify social media use [5, 21, 36]. Such measures may be insufficient to capture how people integrate social media into their daily lives, routines, and their emotional attachment to the platform [6, 21, 36]. Nonetheless, the present study expands the literature available on this topic as limited studies include social media-related instruments at the population level.

Results from this study also suggest that there may be a negative relationship between constant social media use and poor mental health among older adults. These findings showed social media use be particularly harmful for older adults’ mental health, but other factors may be stronger predictors for severe psychological distress. The possibility that those experiencing severe levels of psychological distress or loneliness, turn to social media, or that the distressing events unfolding during the height of the COVID-19 pandemic and other news may worsen mental health for older adults. Many pandemic-related stressors were a result of being exposed to news and other alarming statistics on social media. For example, a recent study of older adults show that use of social media for COVID-19-related information was associated with more anxiety symptoms [45]. This is in contrast to some research which suggests that social media serves as a vehicle for social support for older adults. While more work is certainly needed to ascertain these patterns of moderation on mental health indicators, social media use has elements of social resource building that may offset the potentially harmful or stressful experience of using social media constantly. Considering increased utilization of social media among older-aged adults, social media may still be an important resource for obtaining health information, managing health issues, and building social connections [31].

### Mental health disparities among Asian older adults

It also deserves mention that our study finds that older adults identifying as Asian, had higher odds of experiencing severe psychological distress, and remained significant in our final model controlling for covariates, including loneliness and health status. There are very limited research that solely focus on older adult Asian American mental health issues, particularly disaggregated data by Asian sub-populations [19]. Previous research has yielded inconsistent results about the prevalence of mental health disorders among different ethnic groups of older Asians [19, 20]. Structural factors, including racism or discrimination, to influence disparities in poor mental health among older adult Asian Americans [24]. Since the onset of the COVID-19 pandemic, anti-Asian racist incidents (e.g., verbal harassment, physical assault, property vandalism) have increased dramatically, with many attacks that targeted older immigrants [24]. This finding contrasts against studies which find that Asian groups have better mental health when compared to the general population. Our findings suggest disparities exist among older adults who identify as Asian, and that further work is needed to identify public health policies and strategies to increase Asian older adults’ access to mental health care.

### Future research and policy implications

This study deepens our understanding of the complex interplay between loneliness, social media use, and mental health among older adults. It signals the need for more nuanced measures of social media use, beyond mere frequency and duration, to capture its role and influence on older adults' lives. The study also highlights the need for longitudinal research designs to unravel the causal relationships between these factors, as well as cohort effects. Furthermore, it emphasizes the importance of disaggregating data to understand the unique mental health experiences of diverse ethnic groups, particularly Asian older adults, a group often overlooked in research.

The significant association of loneliness and severe psychological distress among older adults, independent of sociodemographic factors, underscores the urgent need for public health policies aimed at mitigating social isolation in this population. Policies could focus on designing programs that foster genuine social connections and support networks for older adults, with a particular emphasis on those who identify as female, Asian, single, and from lower socioeconomic backgrounds. In light of the findings on social media use, it is crucial for policy to address the potential negative impacts of constant social media use on older adults' mental health. This could involve public awareness campaigns about the potential risks of overuse and the importance of discerning the quality of online information, particularly in stressful situations like a pandemic. The unique vulnerability of older adults identifying as Asian to psychological distress calls for public health policies aimed at reducing racial disparities in mental health care. This could involve culturally sensitive mental health services, anti-racism initiatives, and policies aimed at increasing access to mental health care for this population.

## Limitations

Like all studies, our work is characterized by several limitations. The CHIS is a cross-sectional design and causality cannot be derived from these findings. It is also conceivable that those who have severe psychological distress might be more likely to be lonely. Moving forward, longitudinal data can also provide researchers the opportunity to track any long-term effects of the pandemic on mental health and examine the role of social media as a social connection resource addressing subjective social isolation as the pandemic evolves. The use of cross-sectional data also prohibits us from disentangling age from cohort effects in the analysis. Majority of the sample had healthcare coverage (over 99%) and therefore was not included in the final model. Findings might not be generalizable beyond the state of California. However, given that this study is based on a large sample and state-wide representative data, results of our study may be generalizable to other older adult populations residing in states of similar characteristics.

## Conclusion

Our study adds to existing literature on loneliness, social media use, and mental health among older adults within the context of the COVID-19 pandemic. Our main findings show that loneliness is a strong predictor for severe psychological distress among older adults, despite their level of social media use. While constant social media use was itself associated with severe psychological distress, findings suggest that levels of social media use did not strengthen or worsen the relationship between loneliness and poor mental health. Mixed findings on the association of social media use on mental health exist, suggesting there is a need to further explore specific mechanisms of social media use on mental health. Particularly concerning is our finding that racial and ethnic mental health disparities exist for Asian American older adults, warranting strategies to understand better the impact of COVID-19 pandemic on older adult Asian Americans and improving access to mental health screening and care delivery.

### Supplementary Information

Below is the link to the electronic supplementary material.

Supplementary file1 (DOCX 13 KB)

## Data Availability

CHIS data is publicly available.
