# Supplementary material for: Social isolation, social media use, and poor mental health among older adults, California Health Interview Survey 2019–2020
Source: Soc Psychiatry Psychiatr Epidemiol. 2023 Sep 20;59(6):969–77. doi: 10.1007/s00127-023-02549-2 (PMC11116239; doi:10.1007/s00127-023-02549-2)
Supplement: Supplementary file 1 — Supplementary file1 (DOCX 13 KB) [file 127_2023_2549_MOESM1_ESM.docx]

Appendix. Interaction term results for loneliness x social media use among California older adults.

| **Variable:**  **Loneliness** | **Variable:**  **Level of social media use** | **Crude OR [95% CI ]** |
| --- | --- | --- |
| **Not lonely**  **Lonely**  **Lonely**  **Lonely** | Less than few times  Almost always  Many times  A few times | Ref  0.26 (0.01-1.70)  0.29 (0.02-3.80)  0.46 (0.06-3.50) |
